# Protocol for analyzing immune cell infiltration in 3D pancreatic heterotypic spheroids composed of tumor cells and cancer-associated fibroblasts

**DOI:** 10.1016/j.xpro.2026.104383

**Published:** 2026-02-23

**Authors:** Bessede Thomas, Vezzio-Vie Nadia, Freixinos Clara, Pirot Nelly, Bonnefoy Nathalie, Gongora Celine, Larbouret Christel, Gros Laurent

**Affiliations:** 1 IRCM, University Montpellier, Inserm, ICM, Montpellier, France

**Keywords:** Cell Biology, Cancer, Immunology

## Abstract

We present a protocol to generate and analyze 3D cancer models composed of tumor cells, cancer-associated fibroblasts (CAFs), and immune cells that mimic the complexity of the pancreatic ductal adenocarcinoma (PDAC) stroma. The protocol includes steps and conditions for establishing 3D co-cultures, embedding for histological analysis, and flow cytometry analysis for the immunophenotyping and characterization of tumor-infiltrating immune cells. These models are valuable for testing strategies and studying the roles of CAFs and immune cells in tumor growth, chemoresistance, and immunosuppression.

## Highlights

• Guidelines to construct heterotypic 3D models that mimic PDAC tumor microenvironment
• Steps for analyzing quantitative and qualitative immune cell infiltration
• Procedures to perform spheroid inclusion, sectioning, and labeling for spatial analysis
• Instructions for reproducible manipulation of spheroids to keep a functional structure

## Abstract (editor highlights)

Publisher’s note: Undertaking any experimental protocol requires adherence to local institutional guidelines for laboratory safety and ethics.

## Graphical abstract

**Figure:**
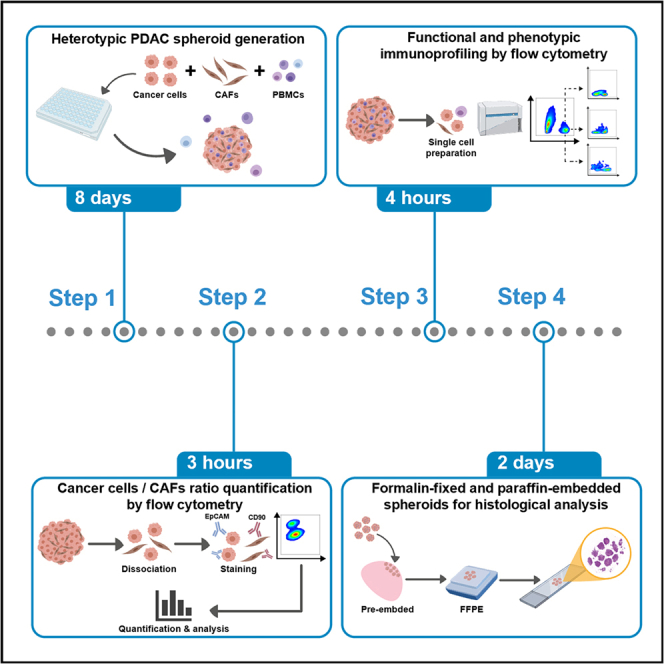

## Before you begin

The tumor microenvironment of pancreatic ductal adenocarcinoma (PDAC) is a complex structure implicated in the resistance to current treatments.^1^^,^^2^ Therefore, the development of new therapeutic approaches to circumvent resistance is a real challenge, particularly because only few in vitro models that mimic the complex interactions in the PDAC ecosystem are currently available. Yet, such models might allow the development of large-scale drug screening to identify new therapeutic strategies.^3^ In recent years, efforts have been made to generate 2D and 3D co-culture models of tumor cells with cancer associated fibroblasts (CAFs), which show very interesting pro-tumor effects. These in vitro models are very relevant,^3^^,^^4^ but they lack an immune compartment to reproduce as closely as possible the tumor microenvironment and the complex crosstalk among the different cell populations.^5^

**Figure 1:**
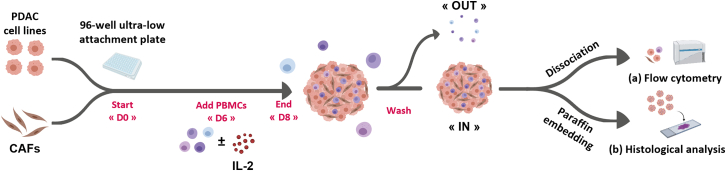
Schematic representation of the protocol to co-culture heterotypic spheroids (PDAC cells and CAFs) with PBMCs from healthy donors At day (D) 0, PDAC cells and CAFs are seeded at a 1:50 or 1:30 ratio (depending on the cancer cell line) in a 96-well clear round bottom ultra-low attachment plate. At day 6 post-seeding, PBMCs (1:10 ratio) and interleukin 2 (IL-2), for immune cell activation, are added. At day 8, spheroids are washed to remove non-infiltrated PBMCs (“OUT”) and are (a) dissociated to analyze the infiltrated PBMCs (“IN”) by flow cytometry, or (b) formalin-fixed and paraffin embedded for histological analysis. Created with BioRender.com.

We present a detailed protocol to generate complex heterotypic 3D models of PDAC (Figure 1). These models reconstitute the complex interactions between cancer cells and stroma components (i.e., CAFs and immune cells). They are composed of PDAC cells (commercial cell lines or established from patient-derived xenografts), primary or immortalized CAFs, and peripheral blood mononuclear cells (PBMCs) isolated from healthy donors. We outline the flow cytometry steps used to determine the cell ratios, an information required to identify the optimal culture conditions, and the immunophenotyping strategy to measure immune infiltration and perform the functional characterization with quantitative accuracy. We also present a detailed method for paraffin embedding spheroids: sample harvesting and preparation, paraffin embedding of several spheroids in each block, section cutting before staining and imaging.

These models, compatible with flow cytometry and histological staining can complement in vivo studies by revealing the cancer cell behavior, microenvironment interactions, and CAF role in tumor growth. They help to understand cancer-related mechanisms, such as chemotherapy resistance and immunosuppression, and are valuable platforms for testing new therapies, including drug combinations that target immune cell infiltration.

### Innovation

This protocol represents a significant methodological advance by establishing an innovative experimental framework based on a triple co-culture system integrating immune cells (PBMCs), primary cancer-associated fibroblasts (CAFs), and pancreatic cancer cells to generate a relevant 3D in vitro model of pancreatic tumor. Few models successfully incorporate key components of the tumor microenvironment (TME). Those described in this article are well suited for studying immune modulation while including CAFs, major players in the pancreatic TME. These conditions reproduce complex CAF-mediated features such as tumor aggressiveness, chemoresistance, and immunosuppression.

The protocol comprises three major components: 1) The generation of heterospheroids, including CAF/tumor cell ratios and final CAF proportions quantification by flow cytometry; 2) Quantitative immunophenotyping and functional characterization of immune cell infiltration into heterospheroids by flow cytometry; and 3) A paraffin-embedding method enabling structural characterization of spheroids through histology. Few in vitro models allow to study the dynamic of immune cell infiltration in relation with both the stroma, and the therapeutic protocols. Major innovations include optimized spheroid dissociation procedures and antibody panels tailored for quantitative phenotypic and functional flow cytometry analysis of immune cell infiltration per spheroid. Finally, our paraffin-embedding method is simple, rapid, and efficient. Unlike most current methods, the one that we describe enables to obtain several spheroids on the same section, providing a significant advantage for reliable and reproducible quantitative analyses.

### Institutional permissions (if applicable)

All the necessary precautions were taken when handling human PBMCs and human cell lines. All experiments involving the use of these materials were conducted in accordance with the Biosafety Level 2 guidelines and using the appropriate protective equipment. CAFs isolated from human PDAC samples were obtained from the Institut du Cancer de Montpellier under the agreement BCB pancreas: ICM 2018-A02901-54 with the informed consent of all patients. Buffy coats for PBMC isolation were obtained from the Établissement Français du Sang under the convention agreement EFS-OCPM: PLER2024-001-R.

## Key resources table

**Table:** 

REAGENT or RESOURCE	SOURCE	IDENTIFIER
**Antibodies**		

Anti-human alpha smooth muscle actin (α-SMA), clone 1A4 (mouse IgG) - ready to use	Roche	760–2833
Anti-human CD107a PE-Cy7, clone H4A3 (Mouse BALB/c IgG1, κ) - dilution [1/200]	BD Bioscience	561348/AB_10644018
Anti-human CD11b FITC, clone M1/70 (Rat IgG2b, κ) - dilution [1/200]	Biolegend	101206/AB_312789
Anti-human CD11c BV421, clone Bu15 (mouse IgG1, κ) - dilution [1/200]	Biolegend	337226/AB_2564485
Anti-human CD14 AF700, clone REA599 (recombinant human IgG1) - dilution [1/200]	Miltenyi Biotec	130-127-384/AB_2904737
Anti-human CD19 BV785, clone HIB19 (mouse IgG1, κ) - dilution [1/200]	Biolegend	302240/AB_2563442
Anti-human CD3 BUV395, clone SK7 (mouse IgG1, κ) - dilution [1/200]	BD Bioscience	564001/AB_2744382
Anti-human CD4 BUV496, clone RPAT4 (mouse IgG1, κ) - dilution [1/200]	BD Bioscience	741134/AB_2870714
Anti-human CD45 BUV661, clone HI30 (mouse IgG1, κ) - dilution [1/300]	BD Bioscience	750178/AB_2874383
Anti-human CD45, clone 2B11 & PD7/26 (mouse IgG1, κ) - ready to use	Roche	760–4279
Anti-human CD56 PE, clone REA196 (recombinant human IgG1) - dilution [1/200]	Miltenyi Biotec	130-113-312/AB_2726090
Anti-human CD8 APC, clone SK1 (mouse IgG1, κ) - dilution [1/200]	Biolegend	344722/AB_2075388
Anti-human CD90 PE-Cy7, clone 5E10 (mouse IgG1, κ) - dilution [1/100]	Biolegend	328123/AB_2561692
Anti-human EpCAM AF700, clone 9C4 (mouse IgG2b, κ) - dilution [1/100]	Biolegend	324244
Anti-human HLA-DR BV650, clone L243 (mouse IgG2a, κ) - dilution [1/200]	Biolegend	307640/AB_2561913
Anti-human interferon-γ BV650, clone 4S.B3 (mouse IgG1, κ) - dilution [1/100]	Biolegend	502538/AB_2563608
Anti-human KI67, clone 30-9 (Rabbit IgG) - ready to use	Roche	790-4286/AB_2631262
Anti-human Pan cytokeratin (PanCK), clone AE1/AE3 PCK26 (mouse IgG1) - dilution [1/4]	Roche	760-2595/AB_2941938
Anti-human PD-1 BV510, clone EH12.2H7 (mouse IgG1, κ) - dilution [1/200]	Biolegend	329932/AB_2562256
Anti-human TCR PAN γ/δ PC5.5, clone immu510 (mouse IgG1, κ) - dilution [1/200]	Beckman coulter	A99021
Anti-human vimentin, clone V9 (mouse IgG1, κ) - dilution [1/2]	Roche	790–2917
Anti-human/mouse granzyme B AF700, clone QA16A02 (mouse IgG1, κ) - dilution [1/100]	Biolegend	372222/AB_2728389
OmniMap anti-rabbit HRP detection kit	Roche	5266548001
Rab Mab: Anti-mouse IgG, clone M204-3 (Rabbit, IgG) – dilution [1/8000]	Abcam	Ab133469/AB_2910607

**Chemicals, peptides, and recombinant proteins**		

0.5% Trypsin-EDTA (10x)	Gibco	15400–054
PROLEUKIN 18 millions U.I (Interleukin-2)	Novartis	Lot n°801313AV
BD GolgiPlug™ Protein Transport Inhibitor (containing brefeldin A)	BD Bioscience	555029
BD GolgiStop™ Protein Transport Inhibitor (containing monensin)	BD Bioscience	554724
Bovine Serum Albumin (BSA)	SIGMA-ALDRICH	A7906-100G
CC1 solution	Roche	950–224
EDTA	Merck	6381-92-6
Eosin (C.I. 45380)	Merck	E4382
100% Ethanol	VWR	83813.36
96% Ethanol	VWR	83804.36
FcR Blocking Reagent, human	Miltenyi Biotec	130-059-901
Fetal Bovine Serum (FBS)	SIGMA-ALDRICH	F7524-500ML Lot: 00016656690
Formaldehyde 4% stabilized, buffered (pH 7.0 ±0.1)	VWR	9713.901
Hematoxylin (C.I. 75290)	Sigma	H3136
Kit Intracellular Staining Buffers	eBioscience	00-5523-00
Paraffin	LEICA	39602012
Paraformaldehyde 16%	Electron Microscopy Science	15710-S
Penicillin/Streptomycin 10 000U/mL	Gibco	15140–122
Trypsin-EDTA (0.05%)	Gibco	25300–054
ViaKrome 808 Fixable Viability Dye	Beckman coulter	C36628
Xylene	VWR	28975.291
0.05% Trypsin-EDTA, phenol red	Gibco	25300054
Anti-Adherence Rinsing Solution	Stemcell	7010
DISCOVERY Inhibitor	Roche	07017944001
Collagenase IV	Merck	C1889-50MG
DMEM/F12 (1:1) (1x) + GlutaMax™-1	Gibco	31331–028
DPBS, no calcium, no magnesium	Gibco	14190–094

**Experimental models: Cell lines**		

Primary CAFs isolated from PDAC samples	Institute of Cancerology of Montpellier	N/A
PANC-1 (KRAS G12D; TP53 R273H)	ATCC	CRL-1469
PancPec (KRAS G12D; MLH1 S252X; PI3K3CA H1047R; BRCA2 G173Vfster12; ChEK2 P552S)	Derived from patient-derived xenografts (PDX) of human primary pancreatic tumor, peritoneum and liver metastasis specimens^6^	N/A
PBMCs from buffy coats of healthy human donors	Établissement Français du Sang	N/A

**Software and algorithms**		

GraphPad Prism version 10.0.0	GraphPad Software	GraphPad Prism
BioRender	BioRender.com	BioRender
FlowJo version 10.10.0	BD Biosciences	FlowJo

**Other**		

300μL Tips, TipOne	Starlab	S1120-9810-C
HistoGel™	Epredia	HG-4000-012
96-well Clear Round Bottom Ultra-Low Attachment Microplates	Corning	7007
Precision Count Beads™	Biolegend	424902

***Note:*** PBMCs were isolated from blood samples of healthy donors using Ficoll and a density gradient centrifugation protocol and stored in liquid nitrogen until use. CAFs were isolated from human PDAC samples using the outgrowth method, as described by Duluc et al.^7^

## Materials and equipment

**Timing: 30 min**

### FACS buffer (500 mL)

• Weigh 1 g of Bovine Serum Albumin (BSA) and transfer to a 499 ml bottle of magnesium- and calcium-free PBS to have a final concentration of 0.2% BSA.
• Add 1 mL of 0.5 M EDTA directly to the bottle to achieve a final concentration of 1 mM EDTA.
• Mix well and store at 4°C for up to 3 months.

***Note:*** FACS buffers typically contain 0.1%–1% BSA to reduce non-specific binding of antibodies and fluorophores to the target cells. To prevent cell clumping, EDTA and Ca^2+^/Mg^2+^-free PBS are used.

### FACS buffer—1% Paraformaldehyde (50 mL)

• Add 3.125 mL of 16% Paraformaldehyde to 46.875 mL of FACS buffer to achieve a final PFA concentration of 1%.
• Protect from light and store at 4°C up to 3 months.

### Spheroid dissociation buffer (extemporaneously preparation)

• Prepare extemporaneously by diluting 10x 0.5% trypsin-EDTA in 1x Ca^2+^/Mg^2+^-free PBS to achieve a final trypsin concentration of 2x.
• Add collagenase IV to a final concentration of 40 μg/mL.

### Interleukin-2 master stock

• Add 1 mL of sterile ultrapure water.
• Add 59 mL of culture medium (DMEM F12 + GlutaMax™, 10% FBS), mix well to achieve a final PFA concentration of 18.3 μg/mL.
• Aliquot into 1 mL tubes, then freeze and store at −80°C for several months.

### Culture medium for 3D spheroid co-cultures and 2D cultures

**Table:** 

Reagent	Final concentration	Amount
DMEM F12 + GlutaMax™	N/A	445 mL
Fetal Bovine Serum	10%	50 mL
Penicillin/Streptomycin 100x	1%	5 mL

• Stored at 4°C for up to 3 months.

### Intracellular staining buffers (kit)

**Table:** 

Reagent	Final concentration	Amount
1x Fixation/Permeabilization buffer (Prepare extemporaneously)	Prepare fresh Fixation/Permeabilization working solution by mixing 1 part of Fixation/Permeabilization concentrate with 3 parts of Fixation/Permeabilization Diluent.	According to the required volume
1x Permeabilization buffer (Prepare extemporaneously)	Prepare a 1X working solution of Permeabilization Buffer by mixing 1 part of 10X Permeabilization Buffer with 9 parts of distilled water.	According to the required volume

### CYTOFLEX LX-UV configuration

All cytometry data were acquired with a Beckman & Coulter CYTOFLEX LX-UV 6 laser. Data were processed and analyzed using the FlowJo v10.10 software. The specific laser/detector configuration is shown in the table below.

**Table:** 

Laser	Filter	Dye
Ultra-Violet (355 nm)	405/30 BP	BUV395
	525/40 BP	BUV496
	675/30 BP	BUV661
Violet (405 nm)	450/45 BP	BV421
	525/40 BP	BV510
	610/20 BP	BV605
	660/10 BP	BV650
	763/43 BP	BV785
Blue (488 nm)	525/40 BP	FITC
	610/20 BP	PEVio615
	690/50 BP	PC5.5
Yellow (561 nm)	585/42 BP	PE
	610/20 BP	mCherry
	675/30 BP	PC5
	710/50 BP	PC5.5
	763/43/BP	PE-Cy7
Red (638 nm)	660/10 BP	APC
	712/25 BP	AF700
	763/43 BP	APCVio770
Infra-Red (808 nm)	840/20 BP	AF790
	885/40 BP	Viakrome 808

### Pannoramic MIDI II scanner

Spheroid sections were scanned using a 3D Histech Pannoramic MIDI II slide scanner. This device is an automatic digital slide scanner with a capacity of 12 slides that can generate digital slides in brightfield and fluorescence.

### ASP300 Leica tissue processor

The Leica ASP300 Tissue Processor is a fully automated system designed for efficient and consistent tissue preparation in high-throughput histology laboratories. It automates critical steps, such as sample dehydration, clearing and paraffin infiltration, ensuring that samples are optimally prepared for downstream applications (e.g., embedding, cutting, and staining).

### Leica Autostainer XL

Section staining and dehydration were performed using the Leica Autostainer XL, an automated platform for high-throughput histology and cytology. It supports various staining protocols, including hematoxylin-eosin-saffron staining, ensuring consistent and reproducible results with minimal manual handling, ideal for high-volume laboratories.

### Ventana Discovery Ultra platform

Histological staining of spheroid sections was performed using the Roche Ventana Discovery platform. This fully automated system is optimized for high-throughput applications, such as immunohistochemistry, *in situ* hybridization and multiplex staining. It ensures reproducible, high-quality results through precise temperature control and reagent delivery, supporting both brightfield and fluorescence imaging. As the platform can process multiple slides simultaneously, it is an excellent choice for large-scale tissue analysis and biomarker research.

## Step-by-step method details

### Generation of heterotypic 3D models with PDAC cells and CAFs

**Timing: 8 days**

The purpose of this first part is to describe an optimized method for 3D culture of human heterotypic spheroids. First, it describes the seeding conditions for the generation of spheroids from human CAFs and pancreatic cancer cell lines (the PDX-derived PancPec cell line and the commercial PANC-1 line) at the optimized ratios of 1:50 or 1:30. Then, at the culture end, flow cytometry is used to determine cell viability, growth and precise cell numbers.

***Note:*** The initial ratio is optimized to generate at the end point spheroids with a diameter of approximately 300–400 μm, 80%–90% of viable, growing cancer cells in the presence of approximately 20%–30% of CAFs. With other cancer cell lines or different time points, the initial ratio must be adjusted and optimized for each cell type.

***Note:*** Only 16 spheroids (e.g., ∼3000–5000 cells per spheroid) are needed to obtain enough cell events for flow cytometry.

***Note:*** Before starting this procedure, ensure that all cell lines are approximately at 50%–70% of confluence in 2D culture flasks. For good cell culture practice, pre-warm the culture medium, PBS, and 0.05% trypsin-EDTA, phenol red to 37°C before use.

**CRITICAL:** CAFs have a much longer doubling time than tumor cells and are less sensitive to overcrowding. We recommend pre-culturing CAFs in T175 flasks to ensure sufficient material. However, during passaging, if diluted too much, these cells may struggle to recover. Therefore, they should not be over-diluted during maintenance. We recommend a dilution ratio of 1:2, or 1:3 at most.

1. Heterotypic spheroid formation (cancer cells + CAFs seeding).
  a. Harvest cells and create a single cell suspension for each cell line.
  b. Aspirate the culture medium from the flask and rinse twice cells with 5 ml (T75 flasks) or 10 ml (T150 flasks) warm Ca^2+^/Mg^2+^-free PBS.
  c. Aspirate carefully the PBS and add 3–5 mL of 0.05% trypsin-EDTA, phenol red (for T75 and T150 flasks, respectively).
    i. Move flask gently to cover all cells with trypsin.
    ii. Incubate cells at 37°C for 5–10 min (10 min for CAFs).
  d. Gently tap the flask and check under the microscope to ensure that all cells have properly detached.
  e. Neutralize trypsin by adding at least an equal volume of culture medium and transfer the cell suspension to a 50 mL conical tube.
    ***Note:*** CAFs are very difficult to detach, it is essential to thoroughly rinse and remove all culture medium traces before trypsin addition. Use pre-warmed trypsin to ensure optimal trypsin activity.
  f. Centrifuge the cell suspensions at 300 x g for 3 min, discard the supernatants, and resuspend pellets with 10 mL of pre-warmed culture medium.
  g. Stain an aliquot of cells with trypan blue to count viable cells in a Malassez chamber (or an equivalent method).
    **CRITICAL:** Cells must be counted at the optimum density to obtain reproducible results. For accurate cell counting, maintain a cell concentration range between 0.5 and 1 million cells per ml. If the cell suspension is too concentrated or too diluted, adjust it and count again.
  h. To confirm that cell count was correct:
    i. Resuspend cells at the concentration indicated in Table 1 and dispense 150 μL of this suspension into three wells of a 96-well ultra-low attachment plate.

**Table 1:** Summary table of cell suspensions

Cell suspension	Cancer cell concentration (cell/ml)	CAF concentration (cell/ml)	Cell number per well/150μL
PancPec alone *(Ratio 1:0)*	333	N/A	50 cancer cells
PancPec + CAFs *(Ratio 1:50)*	333	16 667	50 cancer cells + 2,500 CAFs
PANC-1 alone *(Ratio 1:0)*	1 000	N/A	150 cancer cells
PANC-1 + CAFs *(Ratio 1:30)*	1 000	33 333	150 cancer cells + 5 000 CAFs
    ii. Centrifuge at 300 x *g* for 5 min.
  i. Verify by counting the number of cells in the wells (∼50 cells) using a light microscope.
    **CRITICAL:** To ensure reproducibility, a count of 30 to 60 cancer cells per well is acceptable. If the number of cells is outside this range, adjust the volume of the stock solution to be sampled proportionally. Then, verify the cell count again.
    ***Alternatives:*** If the cell count in the suspension exceeds 50 cells per well (e.g. PANC-1 = 150 cells per well), counting can become difficult. In this case, prepare the cell suspension to deposit 50 cells per well. Verify the accuracy of the count and prepare the cell suspension by multiplying by 3 the stock solution volume to be sampled (e.g. PANC-1 cells)
  j. If the cancer cell count is correct, prepare a new 50 mL Falcon tube for the co-culture seeding by adding the appropriate volume of both cancer cells and CAFs (details provided in Table 1).
  k. Transfer the cell suspension in a reservoir.
    i. Distribute 150 μL/well of seeding cell suspension in a 96-well round bottom ultra-low attachment plate with a multichannel pipette.
      ***Note:*** An electronic multichannel pipette facilitates cell seeding, enhances accuracy, and avoids variability
  l. Incubate without centrifugation at 37°C and 5% CO_2_ for 8 days.
    ***Note:*** In the presence of CAFs, allowing the cells to gently settle without centrifugation helps to form a single, round and homogeneous spheroid per well, preventing the formation of multiple small satellite spheroids within the well.
2. Harvest and dissociation of heterotypic spheroids (here cancer cells + CAFs only).
  a. Using a multichannel pipette with only two 300μL tips:
    i. harvest the desired number of spheroids by aspirating the total volume of each well (2 wells/each time, see Figure 2).

**Figure 2:**
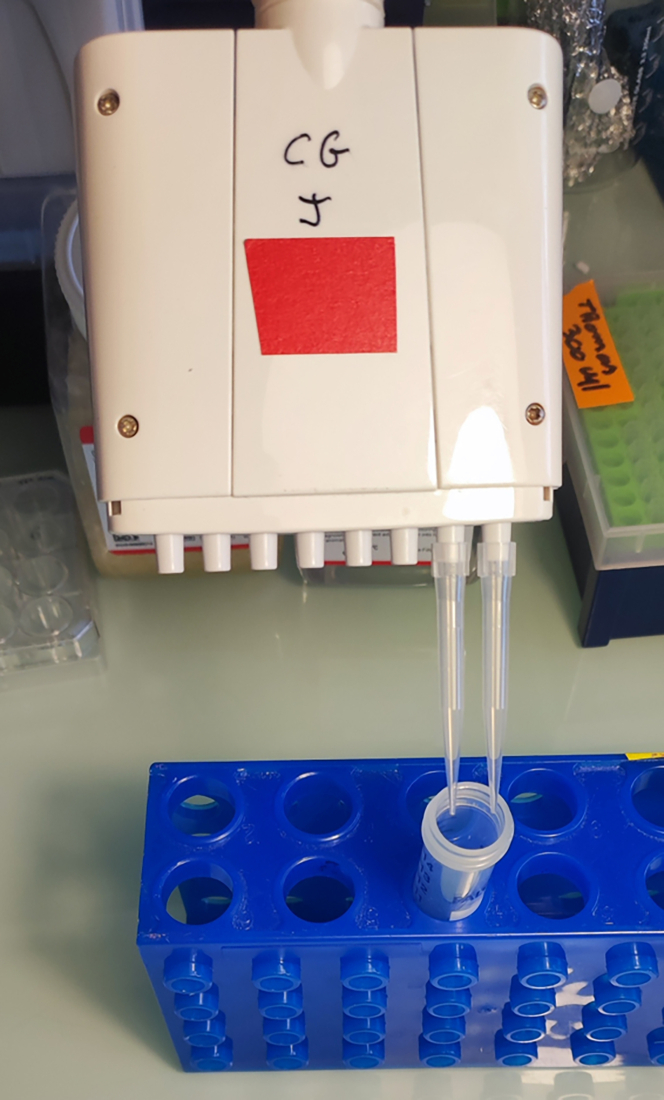
Multichannel pipette with two tips Use only two tips to collect spheroids from wells in pairs.
    ii. Transfer them into a 15 mL centrifuge tube.
      **CRITICAL:** To prevent material loss, check carefully with a lamp because some spheroids may adhere to tips, the bottom of wells, and the walls of plates or centrifuge tube.
  b. Gently allow the spheroids to fall to the bottom of the tube by gravity (approximately 1-3 min), then discard the supernatant by aspiration.
  c. Add 5 mL of 1x Ca^2+^/Mg^2+^-free PBS at 20°C–25°C (room temperature) to rinse and allow the spheroids to sediment again, before aspirating the supernatant.
    **CRITICAL:** Before removing the supernatant, check that all spheroids have sedimented; the sedimentation time may vary depending on the spheroid size and treatment. To avoid aspirating the spheroid pellet, leave approximately 100-200μL of residual volume between each wash. Working with a light helps to better visualize very small spheroids.
  d. Coat 300μL tips by aspirating 200μL of Anti-Adherence Rinsing Solution, then wash the tips by aspirating 200μL of 1x Ca^2+^/Mg^2+^-free PBS.
  e. Transfer in a minimal volume (∼100μL max) all spheroids of one experimental condition to a well of a 24-well plate.
    ***Note:*** Avoid large residual PBS volume in the well because it may dilute the trypsin and affect the quality of the dissociation step.
  f. Count and record the number of collected spheroids per condition using a light microscope or stereoscopic microscope.
  g. Add 500μL of spheroid dissociation buffer (2X trypsin concentrated + 40μg/mL collagenase IV, see material and equipment setup) and incubate at 37°C for 5–10 min.
  h. Flush vigorously with a multichannel pipette using four 200μL tips (see Figure 3) until complete spheroid dissociation.

**Figure 3:**
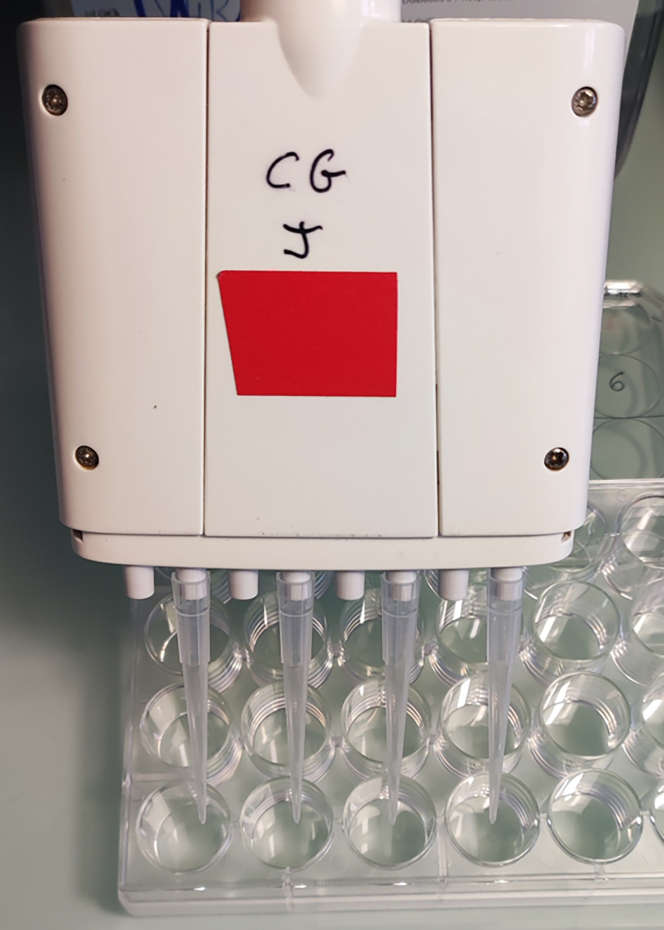
Multichannel pipette with four tips spaced 1 unit apart Use four 200μL tips, spaced 1 unit apart, to aspirate four wells at a time in the 24-well plate.
  i. Check that spheroid dissociation is complete under a light microscope.
    ***Note:*** If dissociation is incomplete, incubate for an additional 3–5 min and repeat step **2h** until complete dissociation.
  j. Add 300 μL of fetal bovine serum (FBS) to the dissociated cell suspension to inactivate trypsin.
3. Quantitative flow cytometry analysis of tumor cell and CAF growth and viability.
  ***Note:*** EpCAM expression can vary across tumor cell lines. Here, PANC-1 cells displayed lower EpCAM expression than PancPec cells. The anti-CD90 antibody is used to distinguish CAFs from other cell populations.
  ***Alternatives:*** If tumor cells do not express EpCAM, another tumor cell marker can be used as substitute.
  a. Transfer the single cell suspension in a 1.5 mL micro tube and centrifuge at 300 x *g* for 5 min.
  b. After sample centrifugation, remove the supernatant and resuspend cells in 100μL Fc block diluted in FACS buffers (dilution 1:100).
  c. Transfer samples to a V-bottom plate and incubate on ice for 30 min.
    i. Meanwhile, prepare the antibody mix (anti-EpCAM, anti-CD90 and LIVE/DEAD viability dye) in FACS buffer (antibody dilutions and volumes are indicated in Table 2).

**Table 2:** Antibody panel for tumor cell and CAF viability and growth analysis

Antibody target	Conjugate	Staining	Volume (μ)^a^
EpCAM	AF700	Extra	1
CD90	PE-Cy7	Extra	1
Live/Dead	Viakrome 808	Extra	0.4

a The specific volume was validated per 0.5∗10ˆ6 million of cells in 100 μL FACS buffer.
    ii. Store the antibody mix on ice and protected from light until use.
  d. Centrifuge the plate at 300 x *g* at 4°C for 3 min, and flip it to discard the supernatant, dry gently on paper towels.
  e. Add 100 μL of the antibody mix to the cells and resuspend by gently pipetting up and down (Table 2).
    i. Incubate on ice in the dark for 30 min.
  f. Wash twice by adding 100 μL of FACS buffer to each well and centrifuge at 300 x g, 4°C, for 3 min. Remove the supernatant by flipping the plate and dry gently on paper towels.
  g. Resuspend the pellet in exactly 150μL of FACS buffer with 1% PFA.
    **Pause point:** Fixed spheroids can be stored in FACS BUFFER-1% PFA at 4°C protected from light for 7 days at most. It is recommended to proceed with the analysis as soon as possible to minimize the loss of antigen signals.
  h. Vigorously vortex the Precision Count Beads™ bottle for 30-40 s to ensure complete mixing and break up of aggregates that may occur during storage.
    i. Add 10 μL of Precision Count Beads™ per sample.
      **CRITICAL:** Accurate pipetting is crucial at 3g and 3h. We recommend using reverse pipetting or an electronic pipette to ensure pipetting the accurate number of beads for each sample.
  i. Prepare the Cytoflex LX flow cytometer (or equivalent) for sample analysis.
    **CRITICAL:** Beads sediment rapidly, and some cytometers do not adequately mix the wells before acquisition. It is recommended to resuspend the samples by pipetting up and down prior to data acquisition. Resuspend every eight wells; a maximum of 15 min delay is tolerated to avoid sedimentation.
  j. Set up the parameters and configure the FSC and SSC channels for the cells of interest as recommended or defined previously, ensuring that the FSC threshold is not too high.
    ***Note:*** Precision Count Beads have a high SSC and low FSC profile. Therefore, the SSC channel needs to be adjusted to make cells optimally visible. This can be done by acquiring a sample of Precision Count Beads alone and comparing it to the cell profile.
  k. Detect the beads in at least one fluorescent channel (see Figure 4). Be sure to adjust the voltage or gain optimally.

**Figure 4:**
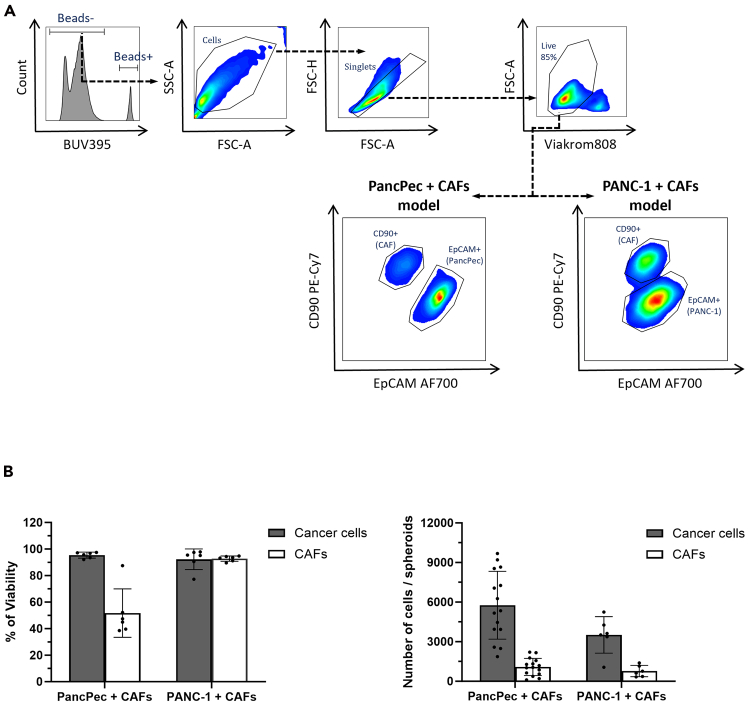
Assessment of the cancer cell/CAFs ratio by flow cytometry after 8 days of 3D co-culture without immune cells (A) Flow cytometry gating strategy for the analysis of CAFs and tumor cells using antibodies against CD90 and EpCAM, respectively (Table 2). Dotted arrows indicate the order of gating. (B) Analysis of cancer cell and CAF viability and counting in spheroids after 8 days of co-culture. Data are represented as mean ± SD.
  l. Proceed with the sample analysis.
    ***Note:*** Precision Count Beads are excited by a variety of lasers, including violet (405 nm), blue (488 nm), yellow/green (562 nm), and red (633 nm).
  m. Use the software statistics function to obtain the bead and cell counts.
    i. You can then calculate the absolute cell counts using the formulas provided in the example below. For further information, please refer to the commercial protocol.

Absolutespheroidcellcount(CellsSpheroids)=(CytometerCellcounta∗beadquantitybCytometerBeadcountc)Numberofspheroidsd

^a^Numerical value of cells counted by the cytometer. ^b^Number of beads added in each well based on the concentration of the commercial solution (e.g., 10 μL of beads at 1×10ˆ6 beads/mL corresponds to 10,000 beads per well). ^c^Numerical value of beads counted by the cytometer. ^d^Number of spheroids counted in step 2f.

### Phenotypic and functional characterization of the immune cell infiltrate in heterotypic spheroids by flow cytometry analysis

**Timing: 4 h for immunophenotyping and 8 h for functional characterization**

This section details the immunophenotyping of infiltrating and non-infiltrating immune cells co-cultured with heterotypic spheroids using flow cytometry. To identify activation markers on immune cell subpopulations (lymphocytes, monocytes, dendritic cells, B cells, natural killer cells, γδ T cells) from PBMCs, we use a panel of specific antibodies (see Table 3). Heterotypic spheroids are co-cultured with healthy donor PBMCs for 2 days at a 1:10 cell-to-PBMC ratio.

***Note:*** Only 24 spheroids (e.g. here ∼150 immune cells per spheroid for the PANC-1 model) are needed to obtain enough cell events for flow cytometry immunophenotyping.

***Note:*** A compensation matrix must be generated for all fluorophores used in the assay to prevent fluorescent signals to spill over between channels. Compensation can be done using compensation beads or cells stained with a single antibody.

***Note:*** To validate the antibody panel and check the compensation, Fluorescence Minus One (FMO) controls must be performed at least once for each staining. FMOs help to define gates, especially when distinguishing positive and negative populations and in case of low expression. Fluorescence spread can occur, especially with brighter fluorophores, and becomes more noticeable after compensation.

4. Addition of PBMCs to the co-culture.
  a. Begin after step 1k after 6 days of incubation.
    ***Note:*** For co-culture with immune cells, PBMCs are added at day 6 at a ratio of 1:10 (CAFs + cancer cells: PBMCs). This means approximately 10 times more PBMCs than the total cell number in the spheroids at day 8. Due to donor heterogeneity, several experiments with different healthy donors are necessary to obtain statistically valid results.
  b. Transfer the cryotube with PBMCs from the liquid nitrogen to a 37°C water bath. Quickly thaw the cryotube in the water bath.
  c. Transfer into a 15 mL centrifuge tube containing 10 mL of culture medium.
    **CRITICAL:** As good PBMC viability is important for the experiments, quickly thawing avoids loss of viability.
  d. Centrifuge cells at 300 x g for 5 min and remove the DMSO-containing supernatants.
  e. Resuspend cells in 10 ml of culture medium and count viable cells in a Malassez chamber (or an equivalent method) after trypan blue staining.
  f. Prepare a cell suspension to distribute 30 000 PBMCs in 50 μL per well, with or without interleukin-2 (IL-2) (10 ng/mL final concentration).
    ***Note:*** Addition of IL-2 ensures immune cell stimulation and enhances infiltration into the spheroids. Other immune cell stimulation methods can also be used.
  g. Transfer in a reservoir and distribute 50 μL/well of PBMC suspension in the 96-well round bottom ultra-low attachment plate with heterotypic spheroids using a multichannel pipette.
  h. Incubate at 37°C and 5% CO_2_ for 2 days (no centrifugation after PBMC addition).
5. On day 8, harvest and dissociate heterotypic spheroids (cancer cells + CAFs + PBMCs).
  a. Collect spheroids using the same method described in step 2a.
  b. Gently allow the spheroids to fall to the bottom of the tube by gravity (approximately 2-3 min), then discard the supernatant by aspiration (non-infiltrated PBMC: OUT).
  c. Add 5 mL of 1x Ca^2+^/Mg^2+^-free PBS at 20°C–25°C (room temperature) to rinse and allow the spheroids to sediment again before aspirating the supernatant.
    i. Repeat this procedure 3 times to remove all non-infiltrated immune cells.
      ***Note:*** A vacuum pump fitted with a glass Pasteur pipette or a plastic serological pipette can be used to easily remove the supernatant.
      **CRITICAL:** Before removing the supernatant, make sure that the spheroids have fully sedimented because sedimentation time varies in function of the spheroid size and treatment. Leave 100–200μL of residual volume during washes to avoid aspirating the pellet. Use a lamp to visualize small spheroids.
  d. Coat 300μL tips by aspirating 200μL of Anti-Adherence Rinsing Solution, then wash the tips by aspirating 200μL of 1x Ca^2+^/Mg^2+^-free PBS.
  e. Transfer in a minimal volume (∼100μL max) all spheroids for the same experimental condition to a well of a 24-well plate.
    ***Note:*** Avoid large residual PBS volume in the well because it may dilute the trypsin and affect the quality of the dissociation step.
  f. Count and record the number of collected spheroids per condition using a light microscope or stereoscopic microscope.
  g. Before starting the enzymatic dissociation, incubate the 24-well plates at 4°C–8°C in a cold room and on ice for 20 min.
  h. Add 500μL of cold spheroid dissociation buffer (2X trypsin concentrated + 40μg/mL collagenase IV, see material and equipment setup).
  i. Incubate at 4°C–8°C on ice in a cold room for 20 min.
  j. Then flush vigorously with a multichannel pipette with four 200μL tips (see photo 2) until complete dissociation of the spheroids in the cell suspension.
    i. Check the complete dissociation under a light microscope (keep on ice).
      **CRITICAL:** The CD4 and CD8 markers are highly sensitive to enzymatic dissociation and can be quickly lost, including intracellularly. To preserve these epitopes, cold incubation helps to stabilize the cell membrane, preventing intracellular depletion and ensuring optimal labeling. After cold incubation, keep samples on ice until trypsin is neutralized.
      ***Note:*** If dissociation is incomplete, incubate for an additional 5–10 min and repeat step **5j** until complete dissociation (Ensure samples are kept cold at all times).
  k. Add 300 μL of FBS to the dissociated cell suspension to inactivate trypsin and transfer each sample to a 1.5 mL micro tube.
  l. Centrifuge cells at 300 x g, 21°C–25°C, for 5 min and discard supernatant.
6. Flow cytometry phenotyping of the immune cell infiltrate.
  a. After sample centrifugation, remove the supernatant and resuspend cells in 100μL Fc block diluted (1:100) in FACS buffer.
  b. Transfer samples to a V-bottom plate and incubate on ice for 30 min.
    i. Meanwhile, prepare the antibody mixture for extracellular staining in FACS buffer (volumes per antibody indicated in Table 3).
    ii. Store the antibody mix on ice and protected from light until use.
  c. Centrifuge the plate at 300 x *g*, 4°C, for 3 min and flip it to discard the supernatant, dry gently on paper towels.
  d. Add 100 μL of the extracellular antibody mix for each condition and mix by gently pipetting up and down. Add 100 μL FACS buffer to the unstained negative control.
  e. Incubate on ice at 4°C in the dark for 30 min.
  f. Wash twice by adding 100 μL of FACS buffer to each well and centrifuge at 300 x *g* at 4°C for 3 min. Remove the supernatant by flipping the plate and dry gently on paper towels.
  g. Fix cells by adding 100 μL of 1x Fixation/Permeabilization Buffer (see material and equipment).
    i. Gently mix the samples by pipetting up and down.
    ii. Incubate in the dark for 20-30 min.
  h. Without washing, directly add 100μL of 1x Permeabilization Buffer.
    i. Centrifuge at 300 x *g* for 3 min.
    ii. Discard the supernatant by pipetting OR flipping the plate and dry gently on paper towels.
    iii. Repeat the wash step with 150μL of 1x Permeabilization Buffer.
      **CRITICAL:** After permeabilization, the pellet becomes transparent and almost invisible. The pellet is more fragile and it is easy to lose material during washing. If the pellet is too fragile, the supernatant can be gently removed after centrifugation by manual pipetting and not by flipping the plate.
  i. Prepare the antibody mix for intracellular staining in 1x Permeabilization Buffer (dilution and volumes per antibody indicated in Table 3).
  j. Stain a second time each condition with 100 μL of the intracellular antibody mix.
    i. Mix by gently pipetting up and down and incubate at 20°C–25°C (room temperature) in the dark for 1 hour.
  k. Wash twice by adding 100 μL of 1x Permeabilization Buffer to each well.
    i. Centrifuge at 300 x *g* at 4°C for 3 min.
    ii. Remove the supernatant by pipetting OR flipping the plate and dry gently on paper towels.
  l. Resuspend the pellets in 150μL of FACS Buffer with 1% PFA.
    **Pause point:** Stained and fixed cells can be stored in FACS buffer-1% PFA at 4°C and protected from light for 7 days at most. It is recommended to proceed with the next steps as soon as possible to minimize loss of antigen signals.
  m. Add 10 μL of Precision Count Beads™ to each sample and run the samples on a Cytoflex LX flow cytometer (or equivalent).
    i. Analyze the data as usual to identify the cell population(s) of interest using the gating strategy shown in Figure 5.

**Figure 5:**
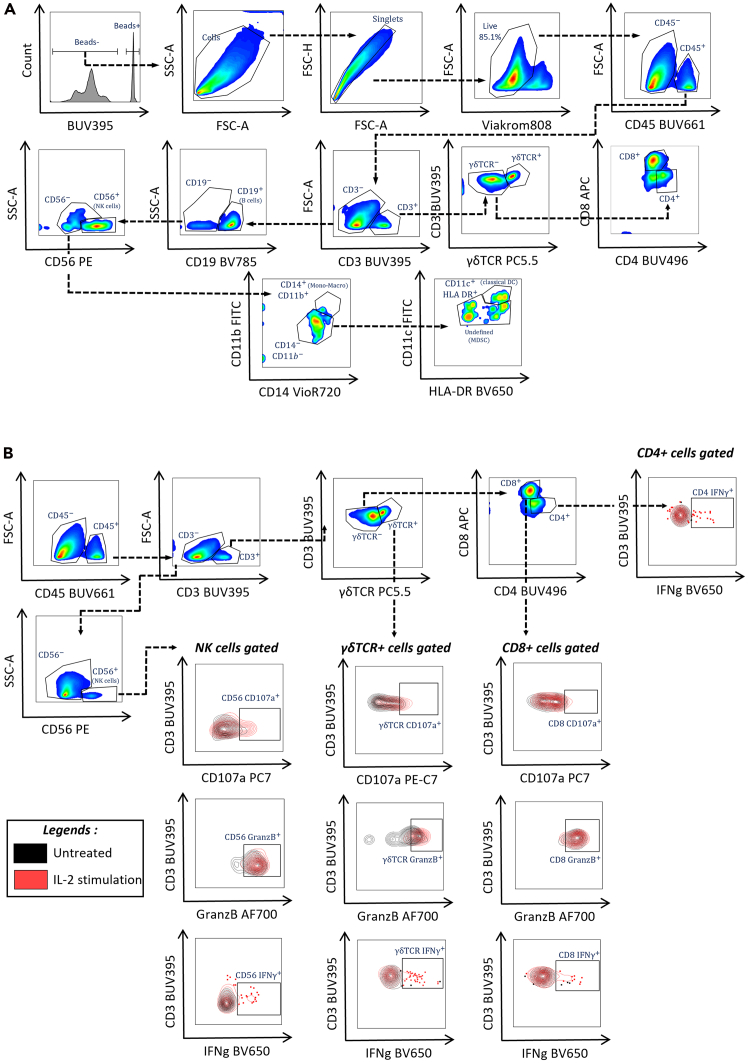
Gating strategy for immunophenotyping the immune cell infiltrate in heterotypic PDAC spheroids by flow cytometry (A and B) Flow cytometry gating strategy for immunophenotyping and functional characterization of different immune cell populations using a panel of specific antibodies (Table 3 and 4). Dotted arrows indicate in which order the gating was performed.
    ii. Quantify the total number of infiltrating immune cells using the same method and formula described in steps 3j, k, l, m.
7. Functional characterization of the immune cell infiltrate by flow cytometry.
  a. Begin after step 4h.
    **CRITICAL:** Here, 192 spheroids per condition (two 96-well plates) are required to obtain a sufficient number of infiltrating immune cells during acquisition. Some markers may be difficult to detect when the number of positive cells is low.
  b. Before spheroid collection, add the BD GolgiStop™ Protein Transport Inhibitor (1:3000) and GolgiPlug™ Protein Transport Inhibitor (1:2000) directly to the wells.
  c. Incubate at 37°C and 5% CO_2_ for 4 h.
    ***Note:*** Cytokines, such as interferon gamma (IFN-γ), are generally rapidly secreted by immune cells and therefore, can be difficult to detect by flow cytometry. The use of protein transport inhibitors allows their accumulation in the cell and significantly increases the ability to detect cytokine-producing cells by immunofluorescence staining. It is recommended not to keep in culture for longer than 12 h after their addition.
  d. Collect spheroids and dissociate them using the methods described in steps 5a, b, c, d, e, f, g, h, I, j, k, l.
  e. After dissociation, proceed with the cell staining steps for flow cytometry using the functional characterization panel described in Table 4 following the procedure described in steps 6a, b, c, d, e, f, g, h, I, j, k, l, m. An example of quantitative and qualitative analysis is given in Figure 6.

**Table 4:** Antibody panel for the functional characterization of PBMCs

Antibody target	Conjugate	Staining^a^	Volume (μ)^b^
CD45	BUV661	Extra	0.33
CD8	APC	Extra and Intra	0.5
CD4	BUV496	Extra and Intra	0.5
CD3	BUV395	Extra	0.5
CD127	BV605	Extra	0.5
γδ TCR	PerCPC5.5	Extra	0.5
CD56	PE	Extra	0.5
CD107a	PECy7	Extra	0.5
PD-1	BV510	Extra	0.5
Live/Dead	Viakrom 808	Extra	0.5
Granzyme B	AF700	Intra	1
IFN-γ	BV650	Intra	1

a Extracellular (extra) or/and intracellular (intra) labeling after permeabilization.

b The specific antibody volume was validated per 0.5∗10ˆ6 million PBMCs in 100 μL FACS buffer.

**Figure 6:**
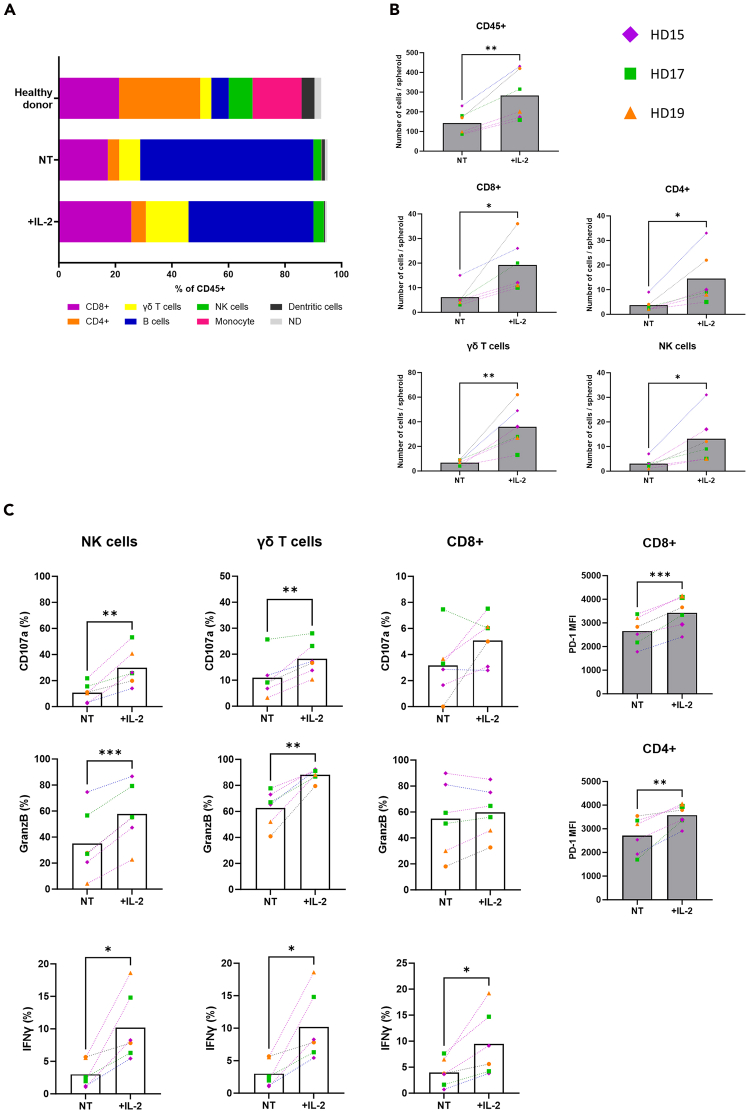
Flow cytometry quantification of immune cell infiltrates and analysis of immune activation marker expression in heterotypic PDAC spheroids (A) PBMC immunophenotyping from healthy donors (HD) and and (B) Quantification of specific immune cell infiltration in heterotypic PANC-1 spheroids following IL-2 stimulation. In heterotypic PANC-1 spheroids, infiltration by immune cells, particularly by effector populations (e.g., γδ t cells and CD8+ T cells), is higher with IL-2 stimulation than without (NT). (C) Analysis of the activation of infiltrating immune cells in heterotypic PANC-1 spheroids incubated or not (NT) with IL-2. After stimulation with IL-2, effector cells show an increase in functional markers (CD107a, granzyme B, IFN-γ) that is associated with an increase in the PD-1 marker on T cells. Data are the mean value of three healthy donors, n = 2 independent experiments; ∗p < 0.05, ∗∗p < 0.01, ∗∗∗p < 0.001, ∗∗∗∗p < 0.0001. (paired Student’s *t* test).

**Table 3:** Antibody panel for immunophenotyping

Antibody target	Conjugate	Staining^a^	Volume (μ)^b^
CD45	BUV661	Extra	0.33
CD8	APC	Extra and Intra	0.5
CD4	BUV496	Extra and Intra	0.5
CD3	BUV395	Extra	0.5
CD14	VioR720	Extra	0.5
CD11c	BV421	Extra	0.5
CD19	BV785	Extra	0.5
CD11b	FITC	Extra	0.5
HLA-DR	BV650	Extra	0.5
γδ TCR	PC5.5	Extra	0.5
CD56	PE	Extra	0.5
CD107a	PE-Cy7	Extra	0.5
PD1	BV510	Extra	0.5
Live/Dead	Viakrom 808	Extra	0.4

a Extracellular (extra) or/and intracellular (intra) labeling after permeabilization.

b The specific antibody volume was validated per 0.5∗10ˆ6 million PBMCs in 100 μL FACS buffer.

### Formalin-fixed paraffin-embedded heterotypic PDAC spheroid samples for histological characterization

**Timing: 8 days**

This section outlines the procedure for formalin-fixing and paraffin-embedding spheroids for histological analysis. It includes detailed steps for embedding spheroids in HistoGel to maximize the number of spheroids sections obtained in the same plane. Topographical staining and immunohistochemistry staining are then performed to visualize different cell populations using specific antibodies.

***Note:*** It is recommended to generate a minimum of 48 spheroids per condition to optimize the number of spheroid sections in the same plane.

**Figure 7:**
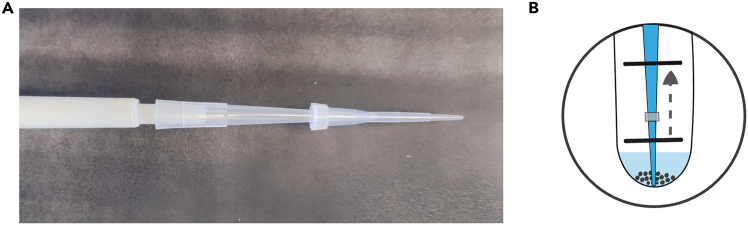
Carefully remove the supernatant for preinclusion in histogel (A) Double tip for removing all supernatant. Using a 200μL micropipette, a 10μL plastic tip is inserted on top of the 200 μL tip to remove carefully the supernatant.
  c. Very gently deposit 150μL of HistoGel, pre-warmed to 65°C, starting on the side of the tube close to the pellet and using a slow, circular movement (see Figure 8).
    **CRITICAL:** Gently apply a layer of HistoGel to the spheroid pellet without disturbing it, ensuring that spheroids remain concentrated at the bottom. To prevent resuspension, add HistoGel slowly. Rewarm HistoGel at 65°C between steps to keep it liquefied because it solidifies quickly. Add HistoGel to one sample at a time.

8. Heterotypic spheroid collection for paraffin embedding (cancer cells + CAFs + PBMCs).
  a. Begin after step 4h (after incubation with PBMC for 2 days).
  b. Collect spheroids using the method described in step 2a.
    ***Note:*** Double-check the plate to ensure that all spheroids have been collected.
  c. Wait few minutes (1–3 min) to allow the spheroids to sink gently to the bottom of the tube by gravity.
  d. Add 5 mL of 1x Ca^2+^/Mg^2+^-free PBS at 20°C–25°C (room temperature) to rinse and allow the spheroids to sediment again, before aspirating the supernatant.
    i. Repeat this procedure 3 times to remove all non-infiltrated immune cells.
      ***Note:*** A vacuum pump fitted with a glass Pasteur pipette or a plastic serological pipette can be used to easily remove the supernatant.
  e. Add 2 mL of 4% formaldehyde and incubate at 20°C–25°C (room temperature) for 1 h.
    ***Note:*** During the fixation period, pre-warm an aliquot of HistoGel to 65°C in a dry bath. It may take up to 45 min to become fully liquefied at this temperature. A 5% concentrated agarose solution may be used at the place of HistoGel. Ensure that the agarose is Low Melting Point Agarose to maintain compatibility during paraffin embedding.
  f. Centrifuge the spheroids at 300 x g for 3 min and discard the supernatant.
  g. Wash once with 5 mL of 1x Ca^2+^/Mg^2+^-free PBS, centrifuge at 300 x g for 3 min, and discard the supernatant. Leave approximately 100-200μL of residual volume for transfer.
  h. Coat wide-orifice pipette tips (300 μL or 1000 μL) with Anti-Adherence Rinsing solution.
    i. Aspirate 200 μL of Anti-Adherence Rinsing solution.
    ii. Rinse the tips with 200 μL of 1x Ca^2+^/Mg^2+^-free PBS.
      ***Note:*** The Anti-Adherence Rinsing Solution prevents spheroids from sticking inside the tips and reduces material loss.
  i. Transfer spheroids with the coated tips into 2.0 mL micro tubes. Try to transfer all spheroids into the smallest possible volume (e.g., 50-100μL max).
  j. Quickly centrifuge the tubes at 300 x g for 1 min to sediment spheroids at the bottom of the tube.
    **Pause point:** Protected from light, fixed spheroids can be stored in PBS at 4°C for several days. However, it is recommended to proceed with the next steps as soon as possible to minimize the loss of antigen signals.
9. Pre-inclusion in HistoGel.
  a. Ensure that the HistoGel is fully liquefied after preheating to 65°C.
  b. Carefully remove the supernatant as much as possible using a double tip (200μL tip + 10μL tip, see Figure 7) taking care not to aspirate the spheroids.
    **CRITICAL:** It is crucial to remove as much liquid as possible to prevent HistoGel dilution and disruption of the gelling process. Ideally, only 10-20 μL of liquid should remain.
    ***Note:*** For large spheroids that do not fit through the orifice of a 10 μL tip, aspirate the liquid by pipetting directly from the bottom of the tube (see photo 3B). This method is not suitable for very small spheroids.

**Figure 8:**
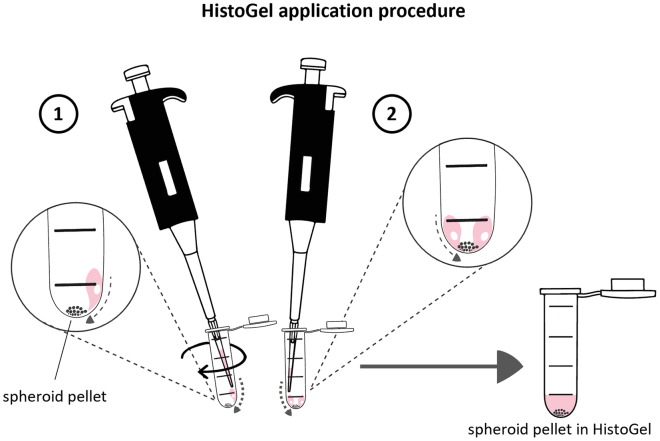
Gentle deposition of HistoGel to preserve the spheroid pellet (1) Begin by gently placing a drop of HistoGel on the inner side of the tube, allowing it to gradually flow toward the sample without disturbing the pellet. Continue to apply drops around the inner circumference of the tube using a circular motion. (2) HistoGel will gradually spread and gently envelop the spheroids, ensuring the preservation of the pellet integrity.
  d. Without delay, perform a quick short spin centrifugation (10 s) at up to 900 x g. Immediately immerse the tubes in ice for at least 5 min.
    **CRITICAL:** A quick centrifugation step helps spheroids to settle at the bottom, aligning them in a single cutting plan. If the spheroid pellet is disturbed during HistoGel deposition (step **9c**), centrifugation may improve their positioning but might not perfectly align them.
    ***Note:*** For the final centrifugation (step 9d), it is preferable to use a centrifuge with an oscillating bucket rotor. This type of rotor keeps the tubes horizontal during operation, helping to position and center the spheroid pellet within the HistoGel dome. If you use a fixed-angle rotor, the pellet may be positioned slightly off-center (see Figure 9). Although this is not critical, it should be taken into account during paraffin embedding to ensure proper sample alignment.
    **Figure 9:**
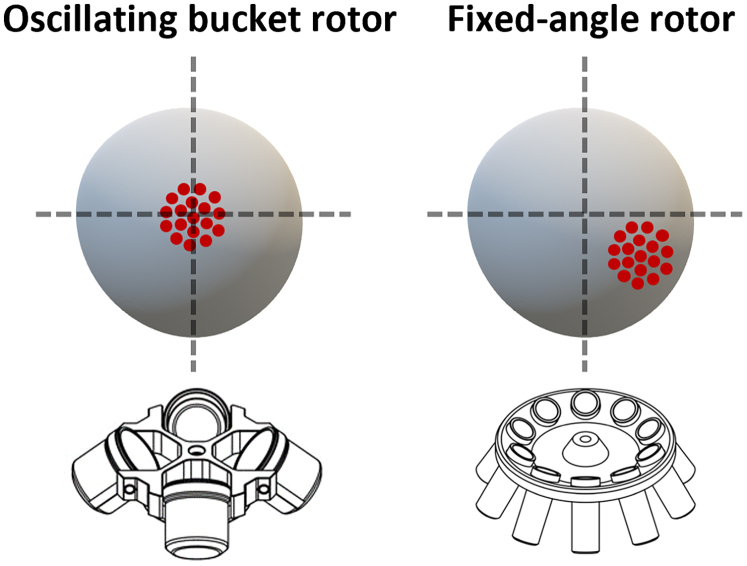
Diagram illustrating the spheroid pellet positioning based on the centrifuge rotor type
    Methods Video S1. Removing the HistoGel block using a fine spatula, related to step 9e
  e. Once the HistoGel has solidified, carefully remove the HistoGel block using a fine spatula (see Figure 10 and Methods Video S1) by gently pushing from the sides of the tube. The dome will invert and can then be removed delicately.

**Figure 10:**
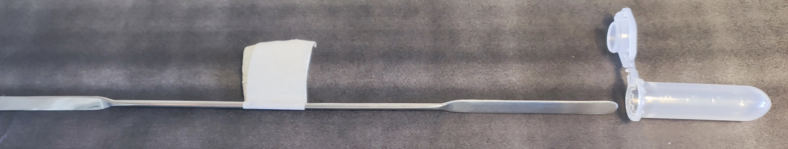
Small rounded-tip spatula Small spatula suitable for insertion into a 2 mL micro tube.
  f. With a scalpel, trim the dome sides to facilitate handling with forceps for inclusion in paraffin (see Figure 11).
    i. Place the dome in an embedding cassette and immerse it in 70% ethanol.
      **Pause point:** HistoGel domes with spheroids can be stored in 70% ethanol at 4°C for several weeks. However, it is recommended to proceed as soon as possible with paraffin embedding.

**Figure 11:**
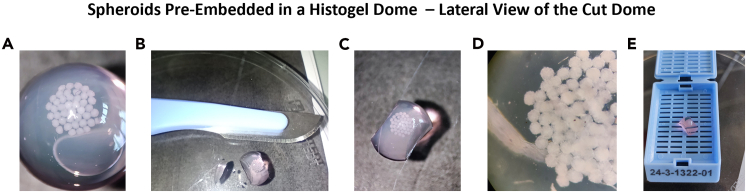
Spheroids pre-included in a HistoGel dome (A) Pre-Inclusion of spheroids in the HistoGel dome: spheroids are positioned on the upper surface of the HistoGel dome. (B) Lateral trimming of the dome with a scalpel: the HistoGel dome containing the spheroids is carefully cut laterally using a scalpel. (C) Lateral view of the cut dome. (D) Micrograph of spheroids in the dome, captured using a binocular microscope. (E) The spheroid-containing dome is placed in an embedding cassette before immersion in ethanol for further processing.
10. Paraffin embedding and cutting steps.
  a. Remove the 70% ethanol.
  b. Transfer the embedding cassettes containing the samples into the baskets of the ASP300 Leica tissue processor.
  c. Impregnating samples with paraffin:
    i. Run the short impregnation program on the Leica ASP300 tissue processor.
    ii. Follow the exposure times to reagents as outlined in the table below:

**Table:** 

Reagents	Time
70% Ethanol	5 min
70% Ethanol	10 min
95% Ethanol	5 min
95% Ethanol	10 min
100% Ethanol	5 min
100% Ethanol	10 min
100% Ethanol	20 min
Xylene	5 min
Xylene	10 min
Xylene	20 min
Paraffin	5 min
Paraffin	10 min
Paraffin	30 min
      **CRITICAL:** If you do not have access to an ASP300 Leica tissue processor, you can manually perform these steps, but the incubation times detailed above should be increased.
  d. Retrieve the HistoGel domes impregnated with paraffin.
  e. Using the embedding station, add liquid paraffin into an embedding mold.
    i. Use forceps to quickly place the domes into the liquid paraffin, ensuring that the pointed end of each dome is oriented downwards, touching the bottom of the mold.
  f. Place the mold on a cold plate to allow the paraffin block to solidify.
    **CRITICAL:** Proper dome orientation in the mold is crucial and is based on the location of the sample. If the sample is slightly off-center, adjust the dome orientation in paraffin to ensure the best possible orientation for cutting sections. The dome is trimmed laterally to facilitate handling with forceps and allow its optimal positioning in the subsequent processes.
    ***Note:*** Solidified FFPE blocks are stored at 20°C–25°C (room temperature) or 4°C for long term.
  g. Cut the blocks in 3 μm thick sections with a microtome, place them on a microscope slide and dry at 37°C for at least 12–16 hours (overnight).
    **CRITICAL:** It is important not to lose the sample during cutting. The position of the spheroid pellet within the paraffin block can vary in depth from one sample to another, and it is easy to quickly use all the sample, if not careful, because of the spheroid tiny diameter (few micrometers). Use a bright light (such as a desk lamp) to precisely locate the spheroids in HistoGel on the paraffin section. Frequently check the sections under the microscope to ensure you have reached the sample and that the cut is satisfactory.
    ***Note:*** As spheroids are very small, you can trim the paraffin around the sample of interest using a sharp lance in a water bath. This technique allows a greater number of sample sections to be placed on the same slide, saving on staining reagents.
11. Hematoxylin, Eosin and Saffron (HES) staining.
  a. Process slides using an automatic stainer (Leica Autostainer XL), running the following HES staining program (Table 5):
    ***Note:*** If you do not have access to an automated stainer, you can manually perform this procedure using separate containers, ensuring that the correct reagents are used and the specified incubation times are followed.

**Table 5:** Program used by the Leica Autostainer XL for HES staining

Reagents	Incubation time
Oven	2 min
Xylene	5 min
Xylene	5 min
100% Ethanol	5 min
96% Ethanol	2 min
70% Ethanol	2 min
Washing with osmosis water	5 min
0.1% Mayer’s hematoxylin	5 min
Washing with osmosis water	10 min
70% Ethanol	5 min
96% Ethanol	5 min
0.25% alcoholic eosin	30 sec
100% Ethanol	5 min
100% Ethanol	5 min
1% Safran	6 min
100% Ethanol	2 min
100% Ethanol	4 min
Xylene	5 min
Xylene	5 min
  b. Mount sections by applying a 1–2 drops of mounting medium (Pertex mounting medium) to each slide, then carefully place a coverslip on top.
12. Antigen retrieval and immunohistochemistry staining.
  a. Using a Roche Ventana Discovery Ultra automated staining instrument (Ventana Medical Systems), configure the immunostaining program using Roche commercial buffers and the specific conditions for each antibody listed in Table 6.
    **CRITICAL:** These staining conditions are tailored for use with a Roche Ventana Discovery automated stainer and the provided buffers and reagents. If you are performing manual immunostaining with different commercial reagents, these conditions may not be optimal. For manual procedures, we recommend to optimize antigen retrieval, primary and secondary antibodies, and other factors in function of your specific reagents and set-up.

**Table 6:** Summary table of the specific programs used by the Ventana automated stainer for immunostaining (single antibody staining)

Primary antibody	Antigen retrieval solution - time - temperature (°C)	Primary antibody dilution – Time – temperature (°C)	Secondary antibody - time - temperature (°C)	Signal enhancement -time	Revelation substrate -time	Counterstaining - time	Post-counterstain -time
**CD45**	CC1 PH8 - 40 min - 95°C	Ready to use - 24 min - 37°C	RabMab 1:8000 in Dako antibody diluent - 60 min – 20°C–25°C	OmniMap Rabbit HRP - 12 min	DAB - 8 min	Hematoxylin −4 min	Bluing Reagent - 4 min
**PanCK**	CC1 PH8 - 32 min - 100°C + protease 3 - 4 min	1:4 in Roche antibody diluent - 32 min - 37°C	RabMab 1:8000 in Dako antibody diluent - 8 min - 20°C–25°C	OmniMap Rabbit HRP - 8 min	DAB - 8 min	Hematoxylin −8 min	Bluing Reagent - 4 min
**α-SMA**	Not necessary	Ready to use - 60 min - 37°C	RabMab 1:8000 in Dako antibody diluent - 60 min - 20°C–25°C	OmniMap Rabbit HRP - 8 min	DAB - 8 min	Hematoxylin −8 min	Bluing Reagent - 4 min
**Vimentin**	CC1 PH8 - 48 min - 100°C	1:2 in Roche antibody diluent - 32 min - 37°C	RabMab 1:8000 in Dako antibody diluent - 8 min - 20°C–25°C	OmniMap Rabbit HRP - 16 min	DAB - 8 min	Hematoxylin −8 min	Bluing Reagent - 4 min
**KI67**	CC1 PH8 – 40 min - 95°C	Ready to use - 32 min - 37°C	N/A	OmniMap Rabbit HRP - 16 min	DAB - 8 min	Hematoxylin −8 min	Bluing Reagent - 4 min
  b. Before starting each protocol apply a deparaffinization step, an endogenous peroxidase neutralization step and a saturation step by incubating the sections in DISCOVERY inhibitors (3% H_2_O_2_) at 20°C–25°C (room temperature) for 8 minutes.
    ***Note:*** Depending on the antibody used, different antigen retrieval programs need to be employed. For example, to reduce background noise for the anti-PanCK antibody, it is recommended to add an enzymatic antigen retrieval step using the reagent Protease 3 from Roche.
  c. Remove slides from the machine and rinse them by immersion in the reaction buffer solution (pH 7.4–7.8).
  d. Dehydrate slides using an automatic stainer (Leica Autostainer XL), running the program shown in Table 7.
    ***Note:*** If you do not have access to an automated stainer, you can manually perform this procedure using separate containers. Make sure that the correct reagents are used and the specified incubation times are followed.

**Table 7:** Program used by the Leica Autostainer XL stainer for slide dehydration

Reagents	Time
95% Ethanol	3 min
100% Ethanol	3 min
100% Ethanol	3 min
Xylene	3 min
  e. Mount sections by applying a 1–2 drops of mounting medium (Pertex mounting medium) to each slide, then carefully place a coverslip on top.
    **Pause point:** Mounted sections after immunohistochemistry staining can be stored at 4°C or at 20°C–25°C (room temperature), protected from light, for several years.
  f. Acquire images using a slide scanner (Pannoramic MIDI II scanner).

## Expected outcomes

Through this protocol, we developed an *in vitro* heterotypic spheroid model of pancreatic adenocarcinoma that mimics the interactions between different cell types found in the tumor microenvironment (CAFs, cancer cells and immune cells). This model allows us to decipher mechanisms in a more physiologically relevant context, particularly those involved in the crosstalk between tumor cells and cells in the tumor microenvironment. This 3D model can be adapted to 384-well plates for high-throughput screening. It is particularly well suited to evaluate the quantitative and qualitative effects of immunomodulatory agents.

**Figure 12:**
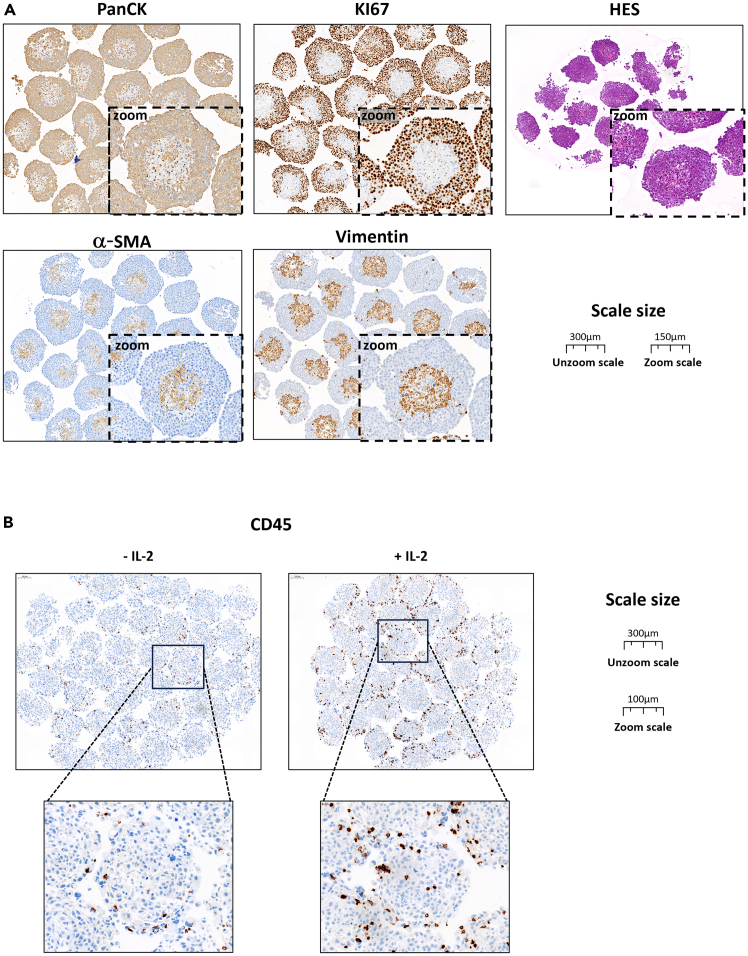
Immunohistochemistry analysis and HES staining of FFPE heterotypic PDAC spheroid sections (A) Immunohistochemistry and HES staining of PancPec + CAF spheroids: the anti-Pan-cytokeratin (PanCK) antibody is used to identify tumor cells, the anti-a-Smooth Muscle Actin (α-SMA) and vimentin antibodies to identify CAFs, and the anti-Ki67 antibody to detect proliferating cells. HES: Hematoxylin, Eosin and Safran staining. (B) Immunohistochemistry analysis of PANC-1+CAF spheroids co-cultured with PBMCs for 48h in presence or absence of IL-2. CD45 staining highlights infiltrating immune cells.

The co-culturing of the three different cell types (Figure 12) allows the rapid formation of stable 3D structures during culture. We developed flow cytometry-based tools to analyze the cancer cell/CAF ratio and easily quantify tumor cell growth (Figure 4). The addition of PBMCs introduces an immune cell component into our model, increasing its complexity and making it more physiologically relevant. Flow cytometry allows the phenotypic and functional characterization of the immune cell infiltration in spheroids (Figure 5). We established a workflow to precisely quantify and characterize immune cell infiltration in the spheroids and designed two antibody panels for the phenotypic characterization of PBMCs (Table 3) and for the functional characterization of immune cells (cytokines and activation markers, such as IFN-γ, granzyme B and CD107a) (Table 4), respectively. The nature of the infiltrating immune cells provides crucial information on the immune cell compartment involvement in the response to treatments. These data will pave the way for exploring combination therapeutic strategies that leverage the synergy with the immune system, particularly in the context of immunotherapy.

As a complementary approach, we have outlined a method for embedding these spheroids in paraffin to perform histologic analyses. This allows us to determine the cellular morphology and 3D architecture and also to stain for specific cell markers (Figure 12). In this method, multiple spheroids are included in a single cutting plane for the image-based quantification and spatial analysis of different cell populations, thus confirming and refining the results obtained by flow cytometry.

This protocol can also be applied to homotypic spheroids composed of pancreatic tumor cells from various cell lines, if they form sufficiently compact spheroids for handling. It is particularly useful to evaluate the effects of treatments in the absence of surrounding cells (CAFs or immune cells) to better understand the specific role of each cell type and their crosstalk in response to treatments. Furthermore, to analyze the influence of the extracellular matrix, spheroids can be embedded in a suitable matrix.

In conclusion, heterotypic spheroids represent a simple, cost-effective, and versatile 3D *in vitro* model for evaluating the efficacy of established and novel therapeutic strategies. This model incorporates essential interactions between cancer cells, CAFs, and immune cells that influence the response to treatments and more accurately reflect the physiological conditions found in pancreatic tumors.

## Limitations

Our *in vitro* 3D heterotypic spheroid model offers the possibility to test new therapeutic strategies for treating patients with pancreatic adenocarcinoma, where cancer cells interact with various cells in the tumor microenvironment. This model can also provide a better understanding of the crosstalk between tumor cells and their environment. However, this model has some limitations. The tumor microenvironment is composed of a wide variety of stromal cells, immune cells and other cell types. To illustrate the concept, this protocol focused only on CAFs and immune cells derived from PBMCs. Other relevant cell types, such as endothelial cells, neural cells and stellate cells, were not included.

The immune cells used in this model are isolated from PBMCs of healthy donors using a Ficoll gradient and present two main limitations: 1) the absence of some myeloid cell types, such as neutrophils and macrophages, and 2) the lack of HLA matching between the different cell populations in the model (allogenicity). To overcome these limitations, when using patient-derived material, we recommend isolating PBMCs or tumor-infiltrating lymphocytes directly from the patient’s blood. This approach would allow HLA matching, thus promoting a more physiological immune response in the context of immunotherapy applications. To include neutrophils, immune cells isolated from fresh whole blood can be used after red blood cell lysis. PBMC-derived monocytes cannot differentiate into macrophages within 48 hours and infiltrate the spheroids.^8^ For a more representative model, we suggest extending the incubation time or directly adding differentiated macrophages.^8^ Lastly, IL-2 is added here to stimulate immune cell infiltration. However, its use may not reflect the clinical reality of pancreatic tumors that are poorly infiltrated by effector cells. Therefore, it is important to consider IL-2 immunomodulatory effects depending on the research objectives.

In this protocol, a single cell ratio was chosen based on the two tumor cell lines (1:50:10 and 1:30:10). The final ratio between CAFs and tumor cells in the spheroids after 8 days of culture was approximately 20% to 80%. However, this ratio is highly dynamic during spheroid development, due to the rapid proliferation rate of cancer cells compared to CAFs, which do not proliferate in 3D culture. Pancreatic tumors are described as having an extremely dense stroma (with up to 80% of CAFs). Therefore, the final ratio obtained in our models may not be representative of human PDACs. The heterotypic spheroids presented here grow in the absence of an extracellular matrix. The stiffness of the extracellular matrix plays a crucial role in tumor initiation and progression by regulating the malignant behaviors of cancer cells.^9^ Therefore, it might be beneficial to use these models in the context of a dense extracellular matrix (e.g., collagen, fibronectin matrix) to achieve a more relevant model.

3D heterotypic models are more relevant *in vitro* models compared with traditional 2D cultures.^10^ They are quick to generate, easy to use, cost-effective and compatible with the screening of different molecules. However, these models do not fully capture the complexity of pancreatic tumors. Therefore, they should be considered as exploratory tools that provide a partial view of the clinical situation. In a research strategy, 3D models are a valuable complementary approach to be used with other models, such as *in vivo* models and organoids.

## Troubleshooting

### Problem 1

Spheroids are difficult to form during culture and tend to develop into multiple spheroids in the same well. Additionally, they are not uniform in size and not properly spherical (related to step 1k).

### Potential solutions

• Centrifugation can disturb spheroid formation, leading to multiple spheroids in the same well. As the effect may vary depending on the cell line, we recommend to test with and without centrifugation to find the optimal method.
• To ensure optimal and homogenous spheroid formation, it is strongly recommended to filter the culture medium and serum to remove debris and impurities that may interfere with spheroid formation.
• Low percentage of methylcellulose in the culture medium (0.5%–4% depending on cell lines) might help the cells to form compact and round spheroids.

### Problem 2

Spheroids become damaged and disaggregate when collected (related to step 2a).

### Potential solutions

• Use tips with an opening larger than the diameter of the spheroids, such as 300μL or 1000μL tips that generally have a wider orifice. You can also use sterile scissors to cut off the tip to enlarge the opening.

### Problem 3

Significant loss of material (spheroids) can occur during the various stages of collection, washing, and transfer (related to step 2a, b, c, e, h; 5c, d, f and 8d, i).

### Potential solutions

• Spheroids tend to easily adhere to tips, the bottom of wells, and the walls of plates or centrifuge tube. Therefore, careful handling and the use of tips coated with Anti-Adherence Rinsing Solution are crucial.
• Using a lamp can help to visualize spheroids.
• Avoid mixing centrifuge tube by inverting or vortexing because this can cause spheroids to stick to the walls.

### Problem 4

Spheroids are not compact enough to be collected by pipetting (related to step 2a).

### Potential solutions

Some cell lines do not form sufficiently compact spheroids to allow harvesting and washing. It is advisable to use cell lines that form compact spheroids for this protocol. However, it is possible to adapt the protocol by using a low percentage of methylcellulose in the culture medium (0.5%–4% depending on the cell line) to help the cells to form compact spheroids.

### Problem 5

Inadequate dissociation of spheroids when trypsin is added (related to step 2h and 5k).

### Potential solutions

• If the residual PBS volume is too large, trypsin may become diluted and take longer to work. When transferring spheroids from the 15 mL centrifuge tube to the 24-well plate, use a small volume (maximum 100 μL). If the PBS volume is too large, you can carefully remove some from the plate without aspirating the spheroids.
• Increase the incubation time and thoroughly flush several times. Incubation time may vary depending on the cell line.

### Problem 6

Immunohistochemistry/immunofluorescence staining issues due to excessively hot HistoGel (related to 9a).

### Potential solutions

• The HistoGel temperature must not exceed 65°C when added to spheroids. If HistoGel is too hot, it may alter the antigens and this can affect the staining quality.

### Problem 7

Spheroids are not properly embedded in HistoGel and escape from the dome during demolding (related to step 9e).

### Potential solutions

• If excessive supernatant remains during step 9b, HistoGel may become diluted and cannot properly encapsulate the spheroids. If spheroids appear to be escaping from the pellet, carefully deposit a drop of HistoGel at the tip of the dome where the spheroids are located to better secure them.

## Resource availability

### Lead contact

Further information and requests for resources and reagents should be directed to and will be fulfilled by the lead contacts, Gros Laurent (laurent.gros@inserm.fr) and Larbouret Christel (christel.larbouret@inserm.fr).

### Technical contact

Further technical information and requests should be directed to and will be fulfilled by the technical contact, Vezzio-Vie Nadia (nadia.vie@icm.unicancer.fr).

### Materials availability

This study did not generate new unique reagents.

### Data and code availability

This study did not generate/analyze datasets/code.

## Acknowledgments

This work was supported by grants from the Ligue Nationale Contre le Cancer, Chocolaterie Gonzalez, the French National Research Agency (ANR) under the Programme Investissement d’Avenir (grant agreement: Labex MabImprove, ANR-10-LABX-53-01) and GIS FC3R. We would like to thank the Etablissement français du sang occitanie for providing Leukopak samples. The MRI SIMCaT platforms are greatly acknowledged. Great thanks to RHEM platform facility for histology techniques and expertise, which is supported by REACT-EU (Recovery Assistance for Cohesion and the Territories of Europe), IBiSA, Ligue contre le cancer, the Occitanie/Pyrénées-Méditerranée, University of Montpellier, and GIS FC3R. The graphical abstract/figures were created using Biorender.com. Thanks to the ICM biological resource center.

## Author contributions

B.T. and V.-V.N. collected the data and performed the analysis. B.T. and V.-V.N. contributed to the design and the analysis of the experiments. V.-V.N., B.T., and C.F. performed the experiments. B.T., V.-V.N., G.C., P.N., B.N., L.C., and G.L. obtained funds and gave advice for the writing of the paper. C.L. and L.G. supervised the work and provided strategic guidance throughout the study.

## Declaration of interests

The authors declare no competing interests.
